# Treadmill intervention attenuates motor deficit with 6-OHDA-induced Parkinson’s disease rat via changes in lipid profiles in brain and muscle

**DOI:** 10.18632/aging.206181

**Published:** 2025-01-03

**Authors:** Binar Panunggal, Tu-Hsueh Yeh, Shu-Ping Tsao, Chun-Hsu Pan, Wei-Ting Shih, Ya-Tin Lin, Amelia Faradina, Chia-Lang Fang, Hui-Yu Huang, Shih-Yi Huang

**Affiliations:** 1 School of Nutrition and Health Sciences, College of Nutrition, Taipei Medical University, Taipei 11031, Taiwan; 2 Department of Nutrition Science, Faculty of Medicine, Diponegoro University, Central Java, Indonesia; 3 Department of Neurology, Taipei Medical University Hospital, Taipei 11031, Taiwan; 4 School of Medicine, Taipei Medical University, Taipei 11031, Taiwan; 5 Ph.D. Program in Drug Discovery and Development Industry, College of Pharmacy, Taipei Medical University, Taipei 11031, Taiwan; 6 Graduate Institute of Metabolism and Obesity Sciences, College of Nutrition, Taipei Medical University, Taipei 11031, Taiwan; 7 Department of Pathology, Taipei Medical University Hospital, Taipei 11031, Taiwan; 8 Research Centre for Digestive Medicine, Taipei Medical University Hospital, Taipei 11031, Taiwan; 9 Neuroscience Research Centre, Taipei Medical University, Taipei 11031, Taiwan

**Keywords:** Parkinson’s disease, 6-hydroxydopamine, treadmill, lipidomic, motor function

## Abstract

One of the key hallmarks of Parkinson’s disease is the disruption of lipid homeostasis in the brain, which plays a critical role in neuronal membrane integrity and function. Understanding how treadmill training impacts lipid restructuring and its subsequent influence on motor function could provide a basis for developing targeted non-pharmacological interventions for individuals living with early stage of PD. This study aims to investigate the effects of a treadmill training intervention on motor deficits induced by 6-OHDA in rats model of PD. PD was induced by injecting 6-hydroxy dopamine (6-OHDA) into the medial forebrain bundle (MFB). For 10 weeks, rats underwent treadmill training on a four-lane motorized treadmill. Motor function deficits were evaluated through behavioral tests. Lipidomic analysis was performed through ultrahigh-performance liquid chromatography-tandem mass spectrometry (UPLC MS/MS). Treadmill intervention significantly improved motor function and restored altered brain and muscle lipid profiles in PD rats. Among the lipid species identified in PD rats, brain abundance was highest for phosphatidylethanolamine (PE), correlating positively with the beam-walking scores; muscle abundance peaked with lysophosphatidylethanolamine (LysoPE), correlating positively with grip strength scores. In the brain, the levels of diacylglycerol (DG), triacylglycerol (TG), and lysophosphatidylcholine (PC) correlated positively with grip strength and rotarod scores, while only phosphatidylethanolamine (PE) linked to beam-walking scores. In the muscle, the levels of phosphatidylinositol (PI), lysophosphatidylethanolamine (PE), lysophosphatidic acid (PA), ceramide (Cer), and ganglioside were positively correlated with grip strength and rotarod scores. In conclusion, treadmill may protect the cortex, mitigating motor deficits via change lipid profiles in the brain and muscle.

## INTRODUCTION

Parkinson’s disease (PD) is the second leading degenerative disorder of the nervous system, following Alzheimer’s disease [1, 2]. PD is characterized by the progressive and selective loss of dopaminergic neurons and the presence of Lewy bodies, manifesting as symptoms such as tremors, rigidity, postural instability, and bradykinesia [1, 3]. Bradykinesia is the primary clinical feature of PD, characterized by a delay in initiating voluntary movements and reduced speed and amplitude of repetitive actions. Other common manifestations include drooling, a lack of facial expression, and micrographia [4]. These motor symptoms typically debut on one side of the body and gradually infiltrate the contralateral side over several years, leaving a devastating impact [5]. Unfortunately, as of now, no definitive treatment exists to arrest the relentless progression of PD. While clinicians have employed various medications, such as dopamine agonists and monoamine oxidase B inhibitors, in PD therapy, their efficacy remains limited [6, 7]. The non-pharmacological treatment for Parkinson’s patients has not yet reached the point of improving lipid metabolism resulting from Parkinson’s disease. Treadmill training is one of the promising non-pharmacological treatments in the future to improve lipid metabolism by restructuring lipid species in Parkinson’s disease.

One promising avenue for managing PD symptoms and potentially modifying its course is through exercise-based intervention [8]. Exercise has been found to protect against neuronal injury, enhance cognitive function, and increase locomotor activity. Exercise correlated to main to the maintenance of redox balance, which is an essential condition for neuronal survival [9]. Interestingly, studies have revealed that aerobic exercise enhances functional connectivity, particularly in the anterior putamen with the sensorimotor cortex relative to the posterior putamen. Additionally, aerobic exercise correlates with heightened functional connectivity within the right frontoparietal network in proportion to fitness improvements, accompanied by a reduction in global atrophy [10]. Therefore, exploring how exercise might influence brain lipid composition and organization could provide valuable insights into its neuroprotective mechanisms. According to animal studies, treadmill exercise and strength can stimulate Sirt1 activity to modulate neuronal inflammation and mitochondrial function through NF-κB (nuclear factor kappa-light-enhancer of activated b cells) deacetylation in 6-OHDA-induced PD rats [11]. Additionally, research involving rat PD models has revealed that treadmill exercise elevates the levels of Nrf2/γ-Glutamate-cysteine ligase catalytic (GLCC) /glutathione and promotes nigrostriatal dopaminergic neurodegeneration [12]. Moreover, it elevates the levels of brain-derived neurotrophic factor (BDNF) and glial-derived neurotrophic factor (GDNF) in the cortex [13]. The activation of BDNF potentially triggers the mechanistic target of the rapamycin (mTOR) pathway, crucial for sphingogenesis, neuronal activation, and axonal myelination, all contributing to enhanced motor learning [14, 15].

Delving into the intricacies of brain lipids, it consists of approximately 50% phospholipids, less than 40% glycolipids, 10% cholesterol, cholesterol ester, and trace amounts of triglycerides. These lipids play pivotal roles in upholding the structural integrity of cell membranes, fostering neural signaling, and facilitating synaptic plasticity. It is worth noting that disruptions in lipid homeostasis have been associated with various neurodegenerative disorders, including PD [16]. Lipidomic studies have indicated that various lipid species are involved in lipid metabolism in patients with PD. Patients with PD have increased levels of specific sphingolipids, such as ceramides, which are associated with neuroinflammation and oxidative stress [17–19]. Ceramides have been implicated in cell death through the binding of death receptors and their ligands (tumor necrosis factor (TNF) and Fas-L) and apoptosis in primary cortical neurons [20, 21]. On a similar note, Lysophosphatidylcholine (LysoPC) emerges as a proapoptotic bioactive, driving apoptosis through the activation of harmful signaling cascades, involving players like the BH3-only protein Bid and caspase-3 [22]. Lysophosphatidylcholine (LysoPC) is also associated with a reduction in the expression of TNF receptor-associated factor (TRAF) 2 [23]. Contrastingly, sphingolipids (SP) reveal a dual role, capable of acting as both proapoptotic and anti-apoptotic secondary messengers, activating various pathways contingent on the cleavage location within the lipid. Both sphingolipids and lysophosphatidylcholine derive from phosphatidylcholine, a versatile mediator serving numerous structural and signaling roles, further implicating them in cellular growth and survival [23].

Notably, exercise significantly influences lipid metabolism, leading to alterations in lipid levels and promoting the utilization of stored fats for energy [10]. Studies in mice have even reported that exercise-induced alterations in brain fatty acids can reduce anxiety levels [24]. This further underscores the profound impact of exercise on lipid metabolism in the brain, yielding bioactive lipids such as sphingolipids and glycerophospholipids that bolster motor and cognitive capacities [17, 25]. Nerve cells utilize dopamine, a brain chemical, to regulate muscle movement. In Parkinson’s disease, the gradual degeneration of dopamine-producing brain cells occurs. The absence of dopamine prevents effective communication from the movement-controlling cells to the muscles, resulting in difficulties in muscle control.

To establish experimental PD, the neurotoxin, 6-OHDA, has been extensively used for reducing the number of nigrostriatal dopaminergic neurons. The neurotoxin impairs motor function and causes dopaminergic cell loss in the pars compacta [26]. In this study, we developed a 6-OHDA-induced PD rat mode and investigated the effect of exercise on motor function in these rats. We further analyzed the effect of low-intensity training on the normalized levels of various lipid species in PD rats by using UPLC MS/MS. This lipidomic study can guide future studies on the role of lipidomic signaling in the development and progression of PD and help identify measurable biomarkers of PD.

## RESULTS

### Quantification of TH-positive dopaminergic neurons

**Figure 1:**
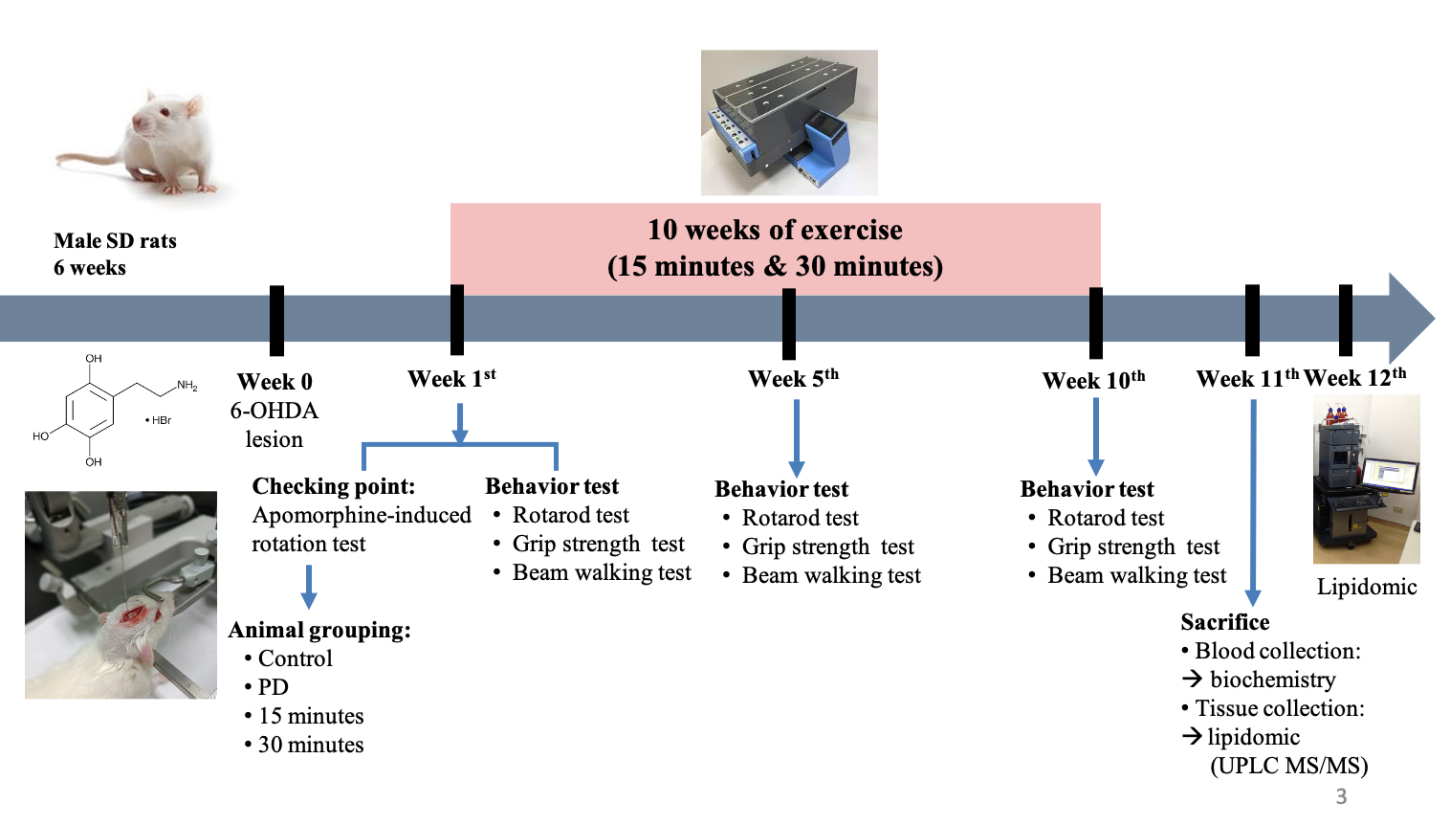
Experimental procedure.

**Figure 2:**
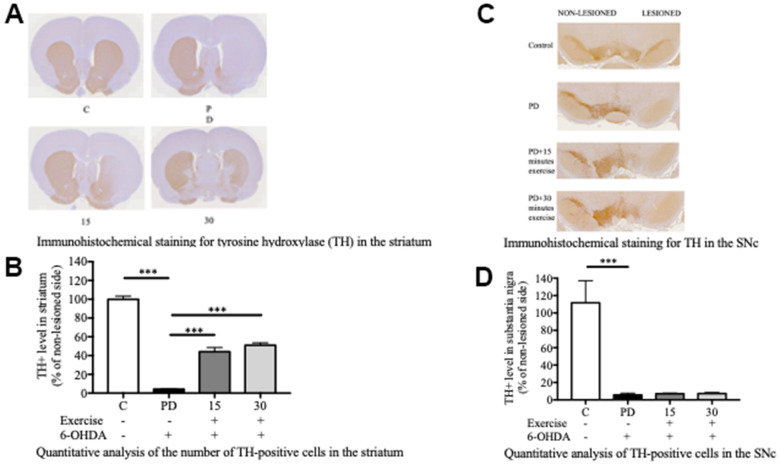
**Exercise rescued the 6-OHDA-mediated reduction of dopaminergic neurons in the substantia nigra pars compacta (SNc) after 10 weeks of exercise treatments.** (**A**) A representative of the immunohistochemical staining for TH (dopaminergic neuron marker) in the striatum. (**B**) Quantitative analysis of the number of TH-positive cells in the striatum. (**C**) A representative of the immunohistochemical staining for the TH in SNc. (**D**) Quantitative analysis of the TH-positive cells in the SNc. Data are presented as the mean ± standard deviation (SD) (n = 5/group). Significance was determined by a one-way ANOVA with Tukey’s post-hoc test. *p < 0.05; **p < 0.01; ***p < 0.001. Control (C), Parkinson’s disease (PD), 6-hydroxy dopamine (6-OHDA).

The establishment of 6-OHDA-induced PD models relies on the fast-acting and selective targeting of dopaminergic neurons by 6-OHDA. In this study, Sprague Dawley rats received a single unilateral injection of 6-OHDA or the vehicle in the striatum and the SNc (Figure 1). Tyrosine hydroxylase (TH)-positive immunoreactivity was evaluated as a marker of viable dopaminergic neurons in the SNc and striatum. All groups varied significantly from the PD group in terms of the number of TH-positive dopaminergic neurons in the striatum (Figure 2A, 2C). By contrast, significant differences were noted only between the control and PD groups in the number of TH-positive dopaminergic neurons in the SNc (Figure 2B, 2D). Treadmill for 15 and 30 min significantly mitigated the loss of TH-positive dopaminergic neurons in the striatum but not in the SNc. These findings suggest that treadmill protects against 6-OHDA-induced inhibition of the dopaminergic nigrostriatal pathway.

### Biochemistry analysis

Table 1 presents the weight of the harvested rat organs and the serum levels of various biochemicals in each group. There were significant differences total cholesterol, triglyceride, and HDL were observed specifically in the serum between exercise and non-exercise groups.

**Table 1:** Effect of exercise on the biochemical characteristic of selected serum and organs in Parkinson’s disease rats.

	**Group**			
	**Control**	**PD**	**15 min exercise**	**30 min exercise**
Body weight (g)	549.6±7.22	423.8±5.03*	486.8±17.0^##^	464.7±16.5^##^
Food intake (g)	28.4±1.11	26.5±0.82*	29.5±0.93	28.9±0.83^##^
Water intake (ml)	45.5±2.72	35.4±0.64*	42.8±2.24	49.9±0.71^##^
**Organ weight**				
Brain (g)	2.20±0.07	2.21±0.09	2.20±0.14	2.20±0.16
Muscle (g)	0.49±0.08	0.39±0.06	0.42±0.06	0.43±0.07
BAT (g)	0.48±0.06	0.57±0.53	0.53±0.16	0.56±0.10
**Brain biochemistry**				
T-Chol (mg/*dL*)	33.9±3.69	37.1±4.06	38.4±1.15	35.5±12.3
TG (mg/*dL*)	11.1±1.22	15.9±3.02	15.1±3.34	13.9±1.58
**Blood biochemistry**				
AST (U/*L*)	152±23.9	143±16.3	162±43.7	134±34.9
ALT (U/*L*)	51.2±10.3	49.1±6.58	53.4±6.19	51.0±5.38
GLU (mg/*dL*)	195±38.6	202±64.9	192±51.4	191±35.0
TP (g/*dL*)	6.73±0.40	6.85±0.67	6.93±0.26	6.82±0.34
ALB (g/*dL*)	4.88±0.23	4.62±0.68	4.96±0.16	4.87±0.30
BUN (mg/*dL*)	13.7±1.40	15.6±3.24	15.7±4.85	12.7±1.18
CREA (U/*L*)	0.24±0.02	0.28±0.03	0.28±0.05	0.30±0.02
TG (mg/*dL*)	107±3.82	39.6±4.81^*^	100±13.4^##^	42.2±18.6^##^
T-Chol (mg/*dL*)	61.9±2.81	29.6±2.95^*^	39.7±9.07^##^	42.2±6.18^##^
LDL (mg/*dL*)	7.22±0.26	4.53±0.23	4.17±1.76	6.73±1.86
HDL (mg/*dL*)	38.2±2.38	11.5±2.03^*^	20.9±3.26^##^	22.3±4.04^##^

Data is presented as mean±SD. *p<0.05 vs. control; #p<0.05 vs. the PD group; ##p<0.01 vs. PD group. Brown Adipose Tissue (BAT), Glucose (GLU), Aspartate Transaminase (AST), Alanine Transaminase (ALT), Total Protein (TP), Albumin (ALB), Blood Urea Nitrogen (BUN), Creatinine (CREA), Triglyceride (TG), Total Cholesterol (T-Chol), Low Density Lipoprotein (LDL), High Density Lipoprotein (HDL).

### Motor function assessment

**Figure 3:**
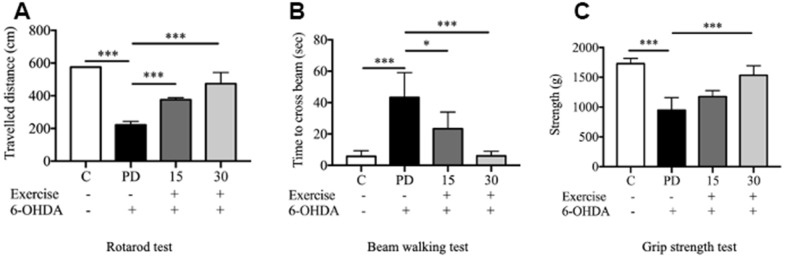
**Assessment of motor impairment.** (**A**) Rotarod test; (**B**) Motor coordination and balance assessed by the beam walking test; (**C**) Grip strength. Data are presented as the mean ± standard deviation (SD) (n = 5/group). Significance was determined by a one-way ANOVA with Tukey’s post-hoc test. *p < 0.05; **p < 0.01; ***p < 0.001. Control (C), Parkinson’s disease (PD), 6-hydroxy dopamine (6-OHDA).

The rotarod test revealed significant between-group differences. Motor deficits were observed in the PD groups. The performance of the PD group in the rotarod test was poorer than that of the control group, as indicated by the small distance covered by the PD rats (*p* < 0.001; Figure 3A). Both 15-minute and 30-min exercises improved the rats’ performance in the rotarod test (*p* < 0.001; Figure 3A).

As indicated by the beam-walking test, 6-OHDA significantly impaired the motor activity of the rats. The PD group required a significantly longer time to cross the beam than did the control group (*p* < 0.001). Both 15-minute and 30-min exercises significantly reduced the beam-walking time, indicating marked improvements in motor activity in the exercise groups (*p* < 0.001) compared with the findings in the PD group (Figure 3B). Furthermore, 6-OHDA significantly reduced the forelimb grip strength (from 1728 to 949.3 g; *t* test. C vs. PD; *p* < 0.001), which was significantly mitigated by 30-min exercise (*p* < 0.001; Figure 3C).

### Body weight (BW), food consumption efficiency, water intake, and body composition

Significant differences in body weight were observed between the untreated group and the treated group. The untreated PD group exhibited lower body weight than the other groups. Furthermore, the treatment of 15 and 30 minutes of exercise during a 10-week period resulted in greater body weight compared to the untreated PD group. This observation suggests that both 15 and 30 minutes of exercise could more effectively mitigate body weight loss compared to the untreated group (Table 1).

Notably, a significant disparity in water and food intakes was identified between the PD group and the 30-minutes of exercise can enhance food intake and ameliorate the body weight loss induced by 6-OHDA, highlighting the potential of exercise to positively impact these physiological parameters (Table 1).

### Lipidomic profiles analysis

**Figure 4:**
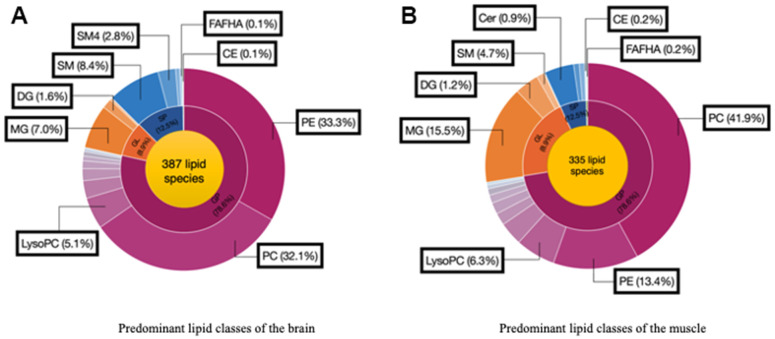
Predominant lipid classes of the (**A**) Brain and (**B**) Muscle. Cholesterol ester (CE), Diglyceride (DG), Fatty acid esters of hydroxyl fatty (FAHFA), Lysophosphatidylcholine (LysoPC), Monoglyceride (MG), Phosphatidylcholine (PC), Phosphatidylethanolamine (PE), Sphingomyelin (SM), Ceramide (Cer).

We analyzed 28 brain and muscle samples, detecting 387 distinct lipid species within the brain and 335 distinct lipid species within the muscle (Figure 4A, 4B). After excluding nonsignificant metabolites, we focused on 38 lipid species belonging to various classes: phosphatidylcholine (PC), phosphatidylethanolamine (PE), phosphatidylglycerol (PG), phosphatidylserine (PS), phosphatidylinositol (PI), phosphatidic acid (PA), cardiolipin (CL), lysophosphatidic acid (LysoPA), lysophosphatidylethanolamine (LysoPE), lysophosphatidylcholine (LysoPC), monoacylglycerol (MG), diacylglycerol (DG), triacylglycerol (TG), ceramide (Cer), sphingomyelin (SM), fatty acid esters of hydroxy fatty acids (FAHFA), and cholesterol esters (CE).

#### Alterations in the brain

**Figure 5:**
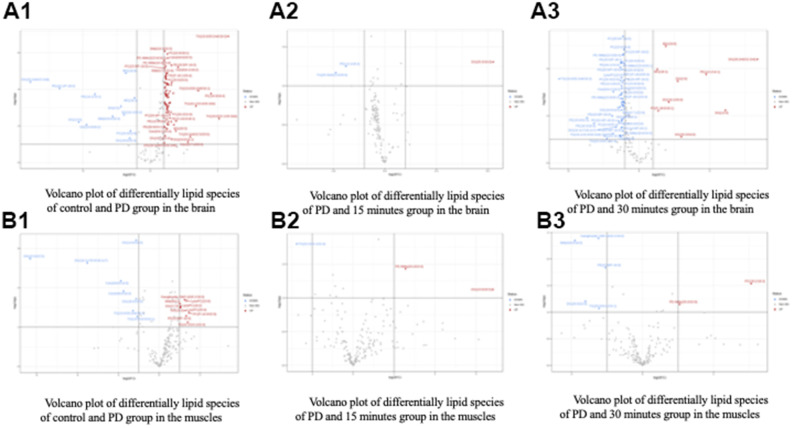
Volcano plot of differentially lipid species of control and treatment group in the (**A**) Brain and (**B**) Muscle. Control (C), Parkinson’s disease (PD).

Alterations in the brain lipid profiles of the two groups are depicted in a volcano plot (Figure 5A) with a *p*-value of <0.05 and a fold change of <1. The levels of DG (20:2n6/0:0/22:2n6), DG (2:0/0:0/0:0), DG (16:1/20:4/0:0), DG (13:0/0:0/0:0), DG(15:0/0:0/20:2), DG (16:1/0:0/20:4), DG (18:1n9/0:0/20:3n6), PE (18:1/P-18:0), PE (14:1/14:1), PE (20:4/P-16:0), PE (14:0/18:0), PE (20:0/24:1), SM (d19:0/20:3), PC (24:0/22:6), Cer (d16:1/20:4), were significantly lower in the PD group than in the control group. Therefore, 6-OHDA significantly reduced metabolite levels in the PD rats.

#### Alterations in the muscle

Alterations in the muscle lipid profiles of the two groups are depicted in a volcano plot (Figure 5B) with a *p*-value of <0.05 and a fold change of <1. The levels of DG (14:0/0:0/16:0), DG (22:6/0:0/22:5), DG (16:1n7/0:0/18:1n7), DG (18:0/0:0/16:0), TG (15:0/20:2n6/18:1), TG (14:0/16:0/16:1), Cer (18:0/14:0), Cer (d18:0/16:0), were significantly lower in the PD group than in the control group. Therefore, 6-OHDA significantly reduced metabolite levels in the PD rats.

Most of the glycerolipids and glycerophospholipids among these differentially expressed lipid metabolites showed a more than ten-fold change between the two groups. These findings suggest that there is an imbalance in lipid metabolism, particularly in the glycerophospholipid and glycerolipid metabolic signalling pathways, in rats with PD (Tables 2, 3). We found that a total of 15 lipid metabolites exhibited differential expression in rats with PD. These metabolites included DG, TG, PC, PE, PG, PI, PS, and Cer.

**Table 2:** Differential lipid brain metabolites expressed under Parkinson’s disease.

**Type of lipid**	**Selected lipid metabolites**	**Fold change**	***p-value***
**Brain**			
Glycerolipids	DG (18:0)	12.793	0.04
	DG (34:2)	3.895	0.03
	DG (36:3 18:2/18:1/0:0)	8.314	0.02
	DG (36:3 20:3/16:0/0:0)	53.766	0.04
	DG (40:6)	52.568	0.04
	TG (16:1/14:0/16:1)	3.312	0.00
	TG (18:1/16:0/20:3)	37.133	0.00
Glycerophospholipids	PC (38:6)	1.554	0.05
	PE (P-36:1)	2.780	0.00
	PE (15:0/22:5)	46.082	0.05
	PG (20:1/18:2)	12.098	0.03
	PIP (22:2)	18.050	0.01
	PS (22:6/22:6)	1.834	0.04
Sphingolipids	Cer (36:1)	2.096	0.03
	Cer (39:7)	1.411	0.00
**Muscle**			
Glycerolipids	DG (18:0)	12.793	0.04
	DG (34:2)	3.895	0.03
	DG (36:3 18:2/18:1/0:0)	8.314	0.02
	DG (36:3 20:3/16:0/0:0)	53.766	0.04
	DG (40:6)	52.568	0.04
	TG (16:1/14:0/16:1)	3.312	0.00
	TG (18:1/16:0/20:3)	37.133	0.00
Glycerophospholipids	PC (38:6)	1.554	0.05
	PE (P-36:1)	2.780	0.00
	PE (15:0/22:5)	46.082	0.05
	PG (20:1/18:2)	12.098	0.03
	PIP (22:2)	18.050	0.01
	PS (22:6/22:6)	1.834	0.04
Sphingolipids	Cer (36:1)	2.096	0.03
	Cer (39:7)	1.411	0.00

DG, diacylglycerol; TG, triacylglycerol; PA, phosphatidic acid; PC, phosphatidylcholine; PE, phosphatidylethanolamine; PS, phosphatidylserine; PIP, phosphatidylinositol phosphate; Cer, ceramide. Lipid metabolites with fold change >10 are highlighted in purple.

**Table 3:** Differential lipid muscle metabolites expressed under Parkinson’s disease.

**Type of lipid**	**Selected lipid species**	**Fold change**	***p*-value**
Glycerolipids	DG (22:6/22:5/0:0)	101.440	0.00
	DG (14:0/18:0/0:0)	1.737	0.04
	DG (14:0/16:0/0:0)	2.489	0.01
	CDP-DG (a-13:0/i-18:0)	7.629	0.00
Glycerophospholipid	PC (P-18:1)	47.193	0.03
	PC (22:0/18:4)	1.927	0.05
	PC (20:4/22:6)	1.705	0.03
	PC (18:1/18:3)	29.987	0.01
	PE (22:6/22:2)	3.008	0.05
	PE-NMe (20:2/22:0)	3.555	0.05
	PE-NMe (16:0/20:3)	1.683	0.03
	PG (i-21:0)	1.643	0.05
	PG (i-12:0/a-17:0)	75.002	0.00
	PG(i-12:0/a-15:0)	119.259	0.01
	PS (20:5/22:0)	5.099	0.05
	PI (16:0/22:2)	1.630	0.03
	PI(16:0/20:4)	2.381	0.02
	PA(20:5/24:0)	1.712	0.02
	PA(20:4/14:0)	32.924	0.01
	LysoPE(P-18:0/0:0)	1.366	0.01
	LysoPA(24:1/0:0)	4.124	0.01
Sphingolipids	Cer(d18:1/18:0)	1.769	0.01
	Cer(d18:0/20:5)	1.535	0.03
	Cer(d18:0/16:0)	16.305	0.01
	Cer(d18:0/14:0)	11.303	0.00
	3-O-Sulfogalactosylceramide (d18:1/24:1)	2.769	0.05
	Ganglioside GM3 (d18:1/18:0)	2.369	0.05
	Ganglioside GM2 (d18:0/26:0)	1.447	0.03

DG, diacylglycerol; TG, triacylglycerol; PA, phosphatidic acid; LysoPA, Lysophosphatidic acid; PC, phosphatidylcholine; LysoPE, lysophosphatidylethanolamine; PE, phosphatidylethanolamine; PS, phosphatidylserine; PIP, phosphatidylinositol phosphate; Cer, ceramide. Lipid metabolites with fold change >10 are highlighted in purple.

Pearson correlation analysis was used to evaluate the relationship between brain and muscle lipid metabolite levels and motor function. The levels of most brain and muscle lipid species were negatively correlated with the rats’ scores in the beam walking test and positively correlated with the rats’ scores in the grip strength and the rotarod test (Tables 4, 5). However, certain lipid species exhibited the opposite results: PE-36:1. In order to find potential lipid biomarkers in PD, we analyzed lipid species that showed significant differences in their levels (P<0.05) and complied them in Tables 2, 3.

**Table 4:** Pearson correlation analysis between differential brain lipids and results of behavior tests.

**Type of lipid**	**Selected lipid species**	**Grip strength (g)**	**Beam walking test (sec)**	**Rotarod Travelled distance (cm)**
Glycerolipids	DG (18:0)	0.54*	-0.38	0.52*
	DG (34:2)	0.49*	-0.44*	0.42
	DG (18:2/18:1/0:0)	0.46	-0.24	0.47*
	DG (20:3/16:0/0:0)	0.62**	-0.65**	0.46*
	DG (40:6)	0.56*	-0.46*	0.52*
	TG (16:1/14:0/16:1)	0.49*	-0.56*	0.55*
	TG (18:1/16:0/20:3)	0.45	-0.51*	0.64**
Glycerophospholipids	PC (38:6)	0.53*	-0.45*	0.59*
	PE (P-36:1)	-0.68**	0.89**	-0.62**
	PE (15:0/22:5)	0.39	-0.46*	0.34
	PG (20:1/18:2)	0.16	-0.09	0.23
	PIP (22:2)	0.25	-0.46*	0.27
	PS (22:6/22:6)	-0.23	0.10	-0.26
Sphingolipids	Cer (36:1)	0.19	-0.35	0.38
	Cer (39:7)	0.32	-0.42	0.20

DG, diacylglycerol; TG, triacylglycerol; PA, phosphatidic acid; PC, phosphatidylcholine; PE, phosphatidylethanolamine; PS, phosphatidylserine; PIP, phosphatidylinositol phosphate; Cer, ceramide. Pearson’s correlation coefficient (r) is presented. The color ranges from dark blue (lowest r-value) to dark orange (highest r-value). *p < 0.05; **p < 0.01.

**Table 5:** Pearson correlation analysis between differential muscle lipids and results of behavior test.

**Type of lipid**	**Selected lipid species**	**Grip strength (g)**	**Beam walking test (sec)**	**Rotarod travelled distance (cm)**
Glycerolipids	DG (22:6/22:5/0:0)	-0.35	0.27	-0.44
	DG (14:0/18:0/0:0)	-0.52*	0.43	-0.51*
	DG (14:0/16:0/0:0)	-0.39	0.32	-0.48*
	CDP-DG (a-13:0/i-18:0)	0.42	-0.40	0.52*
Glycerophospholipid	PC (20:4/22:6)	0.51*	-0.41*	0.57**
	PE (22:6/22:2)	0.45	-0.24	0.57**
	PE-NMe (16:0/20:3)	0.32	-0.39	0.53*
	PS (20:5/22:0)	0.41	-0.36	0.52*
	PI (16:0/22:2)	0.47*	-0.44	0.61**
	PI (16:0/20:4)	0.46*	-0.39	0.59**
	PA (20:5/24:0)	0.36	-0.38	0.52*
	PA (20:4/14:0)	0.32	-0.27	0.36
	LysoPE (P-18:0/0:0)	0.70**	-0.65**	0.63**
	LysoPA (24:1/0:0)	0.61**	-0.44	0.64**
Sphingolipids	Cer (d18:1/18:0)	0.50**	-0.31	0.50**
	Cer (d18:0/20:5)	0.41	-0.45	0.61**
	Ganglioside GM3 (d18:1/18:0)	0.64**	-0.70**	0.67**
	Ganglioside GM2 (d18:0/26:0)	0.45	-0.37	0.64**

DG, diacylglycerol; TG, triacylglycerol; PA, phosphatidic acid; PC, phosphatidylcholine; LysoPE, lysophosphatidylethanolamine; PE, phosphatidylethanolamine; PS, phosphatidylserine; LysoPA, Lysophosphatidic acid; PIP, phosphatidylinositol phosphate; Cer, ceramide. Pearson’s correlation coefficient (r) are presented. The color ranges from dark blue (lowest r-value) to dark orange (highest r-value). *p < 0.05; **p < 0.01.

### Association between lipidomic data and treadmill intervention

**Figure 6:**
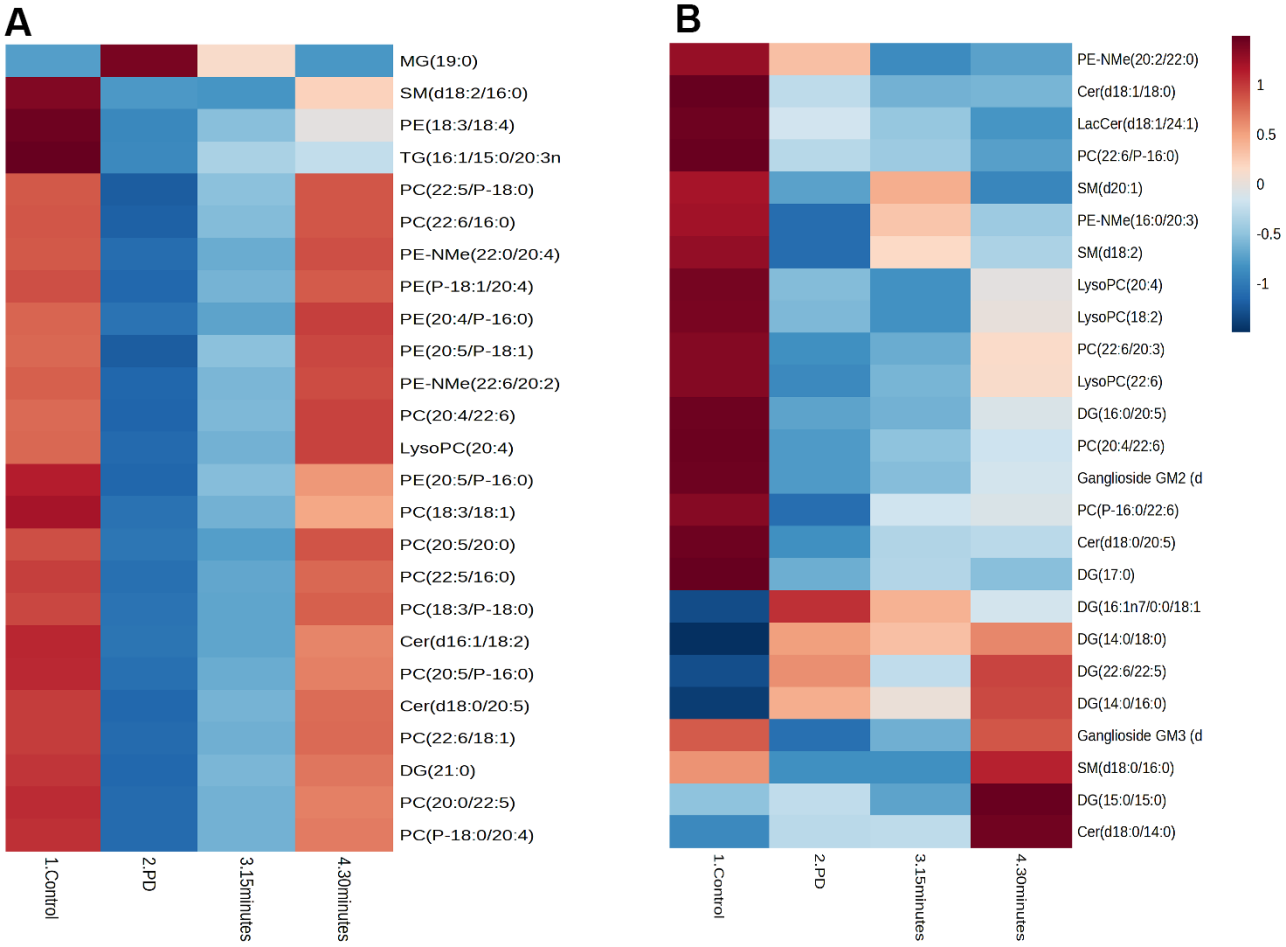
The correlations between identified brain lipid metabolites and clinical parameters in the (**A**) Brain and (**B**) Muscle. Cholesterol ester (CE), Diglyceride (DG), Fatty acid esters of hydroxyl fatty (FAHFA), Lactosylceramide (LacCer), Lysophosphatidylcholine (LysoPC), Monoglyceride (MG), Phosphatidylcholine (PC), Phosphatidylethanolamine (PE), Sphingomyelin (SM), Ceramide (Cer).

**Figure 7:**
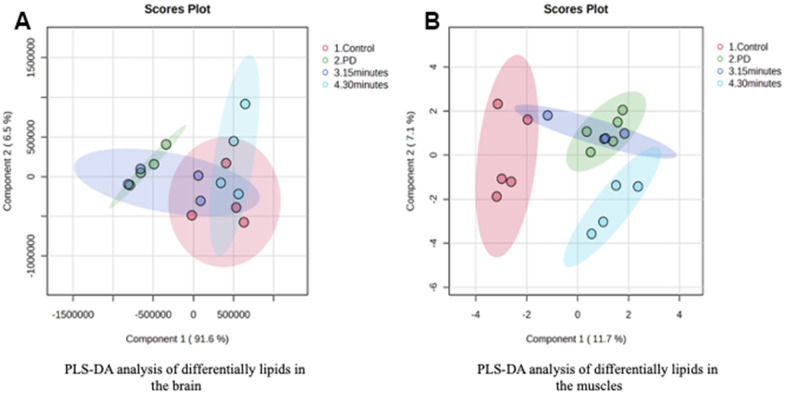
PLS-DA analysis of differentially lipids in the (**A**) Brain and (**B**) Muscle.

**Figure 8:**
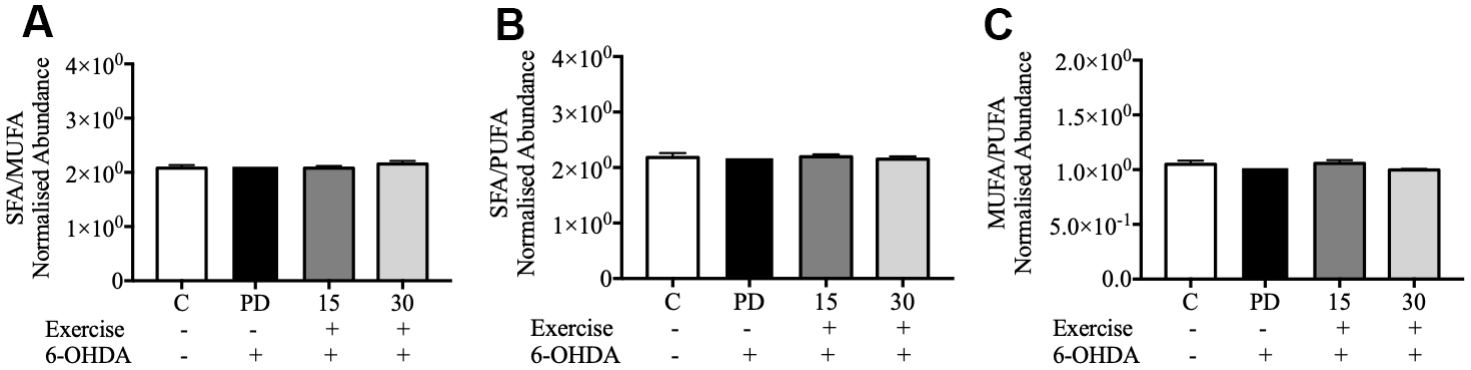
**Relative abundance ratios of fatty acids in the brain.** (**A**) Ratio of SFA to MUFA; (**B**) Ratio of SFA to PUFA; (**C**) Ratio of MUFA to PUFA. Data are presented as the mean ± standard deviation (SD) (n = 5/group). Control (C), Parkinson’s disease (PD), 6-hydroxy dopamine (6-OHDA).

The Pearson correlation analysis indicated a correlation between the selected significant lipid species and treadmill (Tables 4, 5). Among the selected lipidomes, the PD rats mainly accumulated MG (19:0) in the brain and DG (16:1n7/0:0/18:1) in the muscle. Figure 5 presents the lipid species that varied across the groups. Overall, in the brain, there were 387 lipid species, including 82 upregulated and 12 downregulated lipid species (compared with the lipid species of the PD group). In the muscle, there were 335 lipid species, including 12 upregulated and 8 downregulated lipid species (compared with the lipid species of the PD group) were identified in the control, 15-min exercise, or 30-min exercise groups (Figure 5A, 5B). In this study, we observed that the brain samples from the 30-minute group had higher levels of most lipid species (Figure 6A) compared to the other groups. In the muscle, 9 lipid lipids species were elevated compared to the other groups (Figure 6B). The distribution of total lipids across the four groups is depicted in a partial least squares discriminant analysis plot (Figure 7A, 7B), with samples scattered across different areas, indicating different data sets. The ratios of saturated fatty acids (SFA) to monounsaturated fatty acids (MUFA), SFA to polyunsaturated fatty acids (PUFA), and MUFA to PUFA highlight the differences in fatty acid composition. No differences in fatty acid ratios were observed between the groups (Figure 8).

## DISCUSSION

In this study, we conducted experiments utilizing treadmill exercises to evaluate their effects on lipid restructuring in the brain and muscles and how this restructuring subsequently impacts motor function in Parkinson’s disease (PD). Notably, our lipidomic analysis unveiled significant variations in lipid species among the control group, the treadmill exercise group, and the PD group. These results indicate an aberration in lipid metabolism within the brain and muscles, particularly within these pathways, in rats afflicted with PD. A total of 15 lipid species exhibited distinct expressions, encompassing DG, TG, PC, PE, PG, PI, PS, and Cer. Furthermore, these lipid species in the brain and muscles are closely associated with motor function. Our additional findings demonstrate that both 15- and 30-minute sessions of treadmill exercise have a substantial and statistically significant neuroprotective effect on dopaminergic neurons located in the striatum. These observations suggest the potential of treadmill exercise in alleviating the detrimental consequences of 6-OHDA-induced disruption of the dopaminergic pathway, specifically within the nigrostriatal pathway.

Treadmill exercise for 15 and 30 minutes significantly mitigated the loss of TH-positive dopaminergic neurons in the striatum but not in the SNc, suggesting that treadmill exercise protects against 6-OHDA-induced destruction dopaminergic nigrostriatal pathway. Furthermore, the beam-walking test indicated that 6-OHDA significantly impaired the motor activity of the rats, as the PD group required a significantly longer time to cross the beam compared to the control group (p < 0.001). However, both 15-minute and 30-minute treadmill sessions significantly reduced the beam-walking time, reflecting marked improvements in motor activity within the exercise groups. Exercise has shown promise in ameliorating the symptoms of neurodegenerative diseases such as Alzheimer’s and Parkinson’s diseases [27, 28]. Additionally, studies have revealed significant changes in brain structure following motor training and voluntary exercise [29]. Specifically, a study on rats with 6-OHDA-induced PD reported that 30 minutes of daily treadmill exercise for 21 days significantly upregulated TH expression in the striatum [30, 31]. Furthermore, treadmill exercise limited the distribution of α-synuclein, a protein associated with PD, and mitigated the loss of dopaminergic neurons in the substantia nigra and cortex [32]. Another animal study on Wistar rats with 6-OHDA-induced PD demonstrated that 14 days of treadmill exercise mitigated the loss of dopaminergic neurons in the striatum and reduced oxidative stress. Additionally, treadmill exercise increased the number of cells responsive to TH and dopamine transporter [33]. Exercise has been shown to reduce oxidative stress by stimulating mitochondrial biogenesis and upregulating autophagy and neurological functions. It also upregulates the expression of neurotransmitters such as dopamine [34]. Exercise does not negatively impact the brain in individuals with Parkinson’s disease (PD). On the contrary, it contributes positively to their quality of life by alleviating depressive symptoms and enhancing attention, memory capabilities, and motor function [30]. Moreover, a study conducted on animals demonstrated that prolonged exercise resulted in heightened density of dendritic spines. This effect was observed in the hippocampus granule neurons of the dentate gyrus, CA1 pyramidal neurons, and the cortex of adult rats [35]. Our findings are consistent with a study reporting that exercise improves motor function, balance, and spinal learning ability in PD models [27, 28]. Patients with PD have motor and nonmotor deficits, which prevent them from participating in physical activities [29]. The possible may due to exercise helps individuals with motor deficits [29] by promoted the growth of and the connectivity between neurons in the brain and spinal cord [30]. Exercise can induce the production of various growth factors, such as BDNF, particularly mature BDNF, which plays a key role in the growth and maintenance of neurons and their synapses, enhances neuroplasticity and improves motor performance [31]. On the other hand, a previous study using probiotics supplementation showed significantly delayed motor dysfunction by reducing climbing time, foot slips, and the time to reach the beam in a PD model [32, 33].

Investigating alterations in biochemistry parameters in Parkinson’s Disease (PD) is crucial for gaining insights into its progression. A previous study using an Alzheimer’s disease model showed that long-term treadmill exercise improves Aβ levels in the brain by restructuring lipid metabolism such as phosphoglycerides, sphingolipids, and cholesterol [34, 35]. Aβ is a neurotoxin that plays a role in decreasing cognitive function and impairing growth factors, including BDNF. BDNF is essential for synaptic plasticity to improve motor functions such as learning and memory in Alzheimer’s disease (AD) and Parkinson’s disease (PD) [36]. Our study has yielded significant findings regarding disparities in cholesterol, triglyceride, and HDL levels within the serum, distinguishing between individuals in the treadmill and non-treadmill groups. Specifically, we observed an increase in both total cholesterol and triglyceride levels following 15 and 30 minutes of treadmill exercise. These findings align with prior research, suggesting that higher serum total cholesterol is linked to a reduced risk of Parkinson’s disease. Notably, approximately 25% of total cholesterol is distributed within the brain and nerve tissues, contributing to the maintenance of normal cellular structures and functions [37]. On the contrary, a separate study indicated that hypercholesterolemia, accompanied by elevated triglyceride levels, can trigger the accumulation of immune cells, resulting in the release of pro-inflammatory cytokines and the stimulation of α-synuclein aggregation, an association with PD pathology [38, 39]. Therefore, these collective studies suggest that cholesterol may exert bidirectional effects on PD neurodegeneration, potentially serving as both a protective and harmful factor [39]. Regarding HDL concentrations, our results demonstrated lower levels of HDL in the PD group. However, this finding contradicts previous studies that reported higher HDL levels in individuals with PD compared to the control group. Notably, elevated HDL levels have been correlated with the progression of PD [40].

Lipids play a role in lipid homeostasis in the brain and muscle. Lipids are the main constituent of cellular membranes, part of membrane rafts, protein anchors, and signalling molecules [41]. There are eight different types of lipids, classified as fatty acyls, glycerolipids, glycerophospholipids, sphingolipids, sterols, prenols, saccharolipids, and polyketides [42]. Our finding showed that there are five lipid classes in PD brain and muscle. In the PD brain, we found 387 lipid species and 335 lipid species in the muscle. A prior study investigation that dysregulated phospholipid metabolism can disrupt lipid metabolism via SREBP signalling, potentially contributing to impaired heart function and metabolic dysfunction progression.

Lipids have been implicated in many aspects of PD manifestation. Our results show that there is negative correlation between levels of DG and PD. The results are linier in correlation between DG and scores of beam walking test. A positive correlation was observed between diacylglycerol level and neuromotor in PD (Table 2). In previous study, the elevation levels of DG have been observed in the frontal cortex and primary visual cortex of these patients [43]. Our finding showed that Monoacylglycerol has been demonstrated to protect PD rats against neurodegeneration; monoacylglycerol mitigated dopaminergic neuron loss and improved motor function in mice. Another study indicated that the level of monoacylglycerol in the brain is altered in patients with PD [44]. Monoacylglycerol may have therapeutic potential against PD. We also observed sphingomyelin accumulation in the PD rats. A study reported that sphingolipid levels increase in PD. The alteration in sphingolipid level correlates with α-synuclein abnormality [45]. Sphingolipids are essential in cell signalling, maintaining membrane structure, and lipid metabolism. Dysregulation of sphingomyelin metabolism has been implicated in neuropsychiatric disorders, including Alzheimer’s disease, PD, and depression [46]. The role of sphingomyelin in pathogenesis of PD is still debatable. High sphingomyelin levels observed in our PD group may be attributed to acid-sphingomyelinase 1 and 2 and sphingomyelinase 2. Acidic sphingomyelinase (aSMase) is an enzyme responsible for catalyzing the breakdown of sphingomyelin, a type of sphingolipid, into ceramide and phosphocholine. Dysregulation of sphingomyelin metabolism, often due to changes in aSMase activity, can lead to the accumulation of ceramide and disruption of cellular functions [47, 48]. Ceramide has a negative impact on the electron transport chain (ETC), leading to decreased ATP levels [49]. Additionally, ceramide accumulation promotes ceramide-induced mitophagy. Consequently, sphingolipids directly influence various aspects of mitochondrial function that are also implicated in PD, such as ETC function, mitochondrial remodelling, and mitophagy [25, 50].

Changes in lipid metabolism are increasingly recognized as playing a role in the development of Parkinson’s disease (PD), affecting motor function [51]. Sphingolipids, which encompass sphingomyelin and glycosphingolipids, serve as vital constituents of myelin sheaths. Disturbances in sphingolipid metabolism, such as mutations in enzymes responsible for sphingolipid synthesis or turnover, have the potential to impact the composition and stability of myelin [52]. Myelin, a lipid-rich membrane enveloping axons in the nervous system, serves as insulation and facilitates the rapid transmission of nerve impulses. It promotes efficient communication between neurons by enabling swift propagation of action potentials along axons [53]. Alterations in myelin composition, such as changes in lipid rafts or disruptions in protein-lipid interactions, may contribute to dysfunction of motor neurons [54].

In our study, a positive correlation was observed between the level of phosphatidylcholine, including lysophosphatidylcholine, and the extent of motor function in the PD rats. The level of phosphatidylcholine in the skeletal muscle increases with increasing exercise frequency [55]. Phosphatidylcholine is the most abundant glycerophospholipid in membranes; it regulates inflammation, enhances neuronal differentiation, modulates neuronal plasticity, prevents reduced neurogenesis, and maintains cholesterol homeostasis [56–58]. Our findings revealed reductions in almost all types of phosphatidylcholine in the frontal cortex of the PD rats. Phosphatidylcholine upregulates BDNF expression in the brain cells of rats. The administration of lysophosphatidylcholine to the brain tissues of rats has been demonstrated to increase BDNF levels and improve brain function [59].

## CONCLUSIONS

We observed that 6-OHDA reduced motor function and altered brain and muscle lipid metabolism in PD rats; however, exercise improved the 6-OHDA-induced motor impairment in these rats. The treadmill training influences the remodeling of phospholipids, particularly PC, LysoPC, and PE in 6-OHDA-induced PD-like rats. These findings suggest that treadmill intervention attenuating the PD motor deficit may attribute to the brain and muscle lipid changes.

## MATERIALS AND METHODS

### Materials

Serum specimens were collected to evaluate hepatic, renal, and glycemic functions, and analyses were conducted utilizing the Roche Modular P800 system (Roche Diagnostics, Indianapolis, IN, USA). Lipidomic assessments were performed on cerebral and muscular tissues. Additionally, 6-hydroxydopamine (6-OHDA), apomorphine hydrochloride, and desipramine were acquired for experimental purposes. All reagents employed in this research were procured from Sigma-Aldrich (St. Louis, MO, USA).

### Animals

Fifty-one 6-week-old male Sprague Dawley rats were purchased from BioLASCO, Taiwan. The rats were maintained under a 12 h light/dark cycle and were provided with food and water ad libitum. After 1 week of acclimatization, the rats were randomly divided into the following four groups: control, PD, PD plus 15-min exercise, and PD plus 30-min exercise (Figure 1). To induce PD-like motor deficits in the rats, 6-OHDA was unilaterally injected (details provided in Section 2.3). All experiments were conducted in compliance with the code of practice for the care and use of animals for scientific purposes in Taiwan.

### Establishment of PD through unilateral 6-OHDA injection

The rats were anesthetized with xylazine (1 mL/kg; Soleil), and their heads were held in a fixed position by using a stereotaxic frame (David Kopf Instruments, Tujunga, CA, USA). Then, 6-OHDA (3 μg/μL; total volume, 3 μL) was bilaterally injected into the substantia nigra pars compacta (SNpc) by using a 26-gauge stainless steel needle connected to a 5-μL Hamilton syringe; for the injection, the following coordinates were considered: anterior–posterior, 4.4 mm from the bregma; medial–lateral, 1.2 mm from the midline; and dorsal–ventral, −7.9 mm from the skull. The flow rate (1 μL/min over 3 min) was controlled using an electrical pump (KD Scientific Holliston, MA, USA). A 2–3 cm incision was made. The injection needle was held in place for an additional 5 min to avoid reflux. A rotation test was performed to confirm PD induction in the rats 6 weeks after 6-OHDA injection; for the rotation test, the rats were injected with apomorphine. Rats that performed >7 contralateral rotations/min were considered to have PD.

### Behavioral tests

### Rotarod test

Motor coordination and balance were tested through the rotarod test, which was performed using an accelerating rotarod (7650 Rotarod, Ugo Basile, Collegeville, PA, USA) at 10 rpm for a maximum duration of 2 min. The time spent on the rod was recorded.

### Beam-walking test

The beam-walking test was performed using a previous study with modifications. The beam-walking apparatus consisted of a long strip of wood (length of 100 cm, width of 0.6 cm). The beam was suspended at a height of 60 cm, with the other end attached to an enclosed box. The rats were placed at the initial 20 cm of the beam and were trained to cross the beam to the enclosed box within 60 s. The time to cross the beams and the number of foot slips off the beam in each trial were recorded. For each test day, the average time for a total of four trials was calculated for each rat.

### Grip strength

Muscle strength was assessed using the four-limb hanging and grip strength tests described in a relevant study. Each experiment consisted of three trials, and the mean value of the effort was calculated in grams [60, 61].

### Treadmill running

A four-lane motorized rat treadmill was used for exercise training. The rats in the exercise groups were introduced to the treadmill 1 day before the surgery. The treadmill speed was set at 17 m/min, and the exercise duration was 10 min [11]. A day after the completion of the behavioral test and the confirmation of SNc degeneration, the rats were forced to run on the Treadmill for 15 min twice daily (with a 15-minute interval between two running sessions) or for 30 min per day at a speed of 10 m/min for a total of 30 days.

### Lipid extraction

To isolate lipids, the classical Folch method, with some modifications [62], was employed. In brief, 40 mg of prefrontal cortex tissue, 30 mg of muscle tissue, and 50 mg of brown adipose tissue were homogenized in 300 μL of prechilled phosphate-buffered saline. Each resultant homogenate was mixed with 2 mL of methanol and 3 mL of chloroform and shaken for 1 h. Subsequently, 1.25 mL of deionized water was introduced to the mixture, allowing it to settle for 10 minutes to facilitate phase separation. The samples underwent centrifugation at 3000 ×g for 30 minutes at 4° C, and the organic layer was harvested and dried overnight using a vacuum dryer. For sample preparation, the crude lipids were dissolved in a 250 μL solution of isopropanol/acetonitrile/deionized water (2:1:1 v/v/v).

### Lipidomic analysis

The ACQUITY Premier UPLC system coupled with a quadrupole-time of flight mass spectrometer (SYNAPT G2 High Definition MS System) was used for untargeted lipidomic analysis. The total lipid extract was separated using the ACQUITY UPCLC CSH C18 column; the mobile phase comprised acetonitrile/water and isopropanol/acetonitrile. Gradient elution was performed. The injection volume was 5 μL, and the elution rate was 4 μL/min. Tandem mass spectrometry was utilized to ionize lipids, and the resulting data underwent processing with MassLynx. Following this, Progenesis QI was employed for tasks such as peak alignment, peak picking, adduct deconvolution, and sample-wise normalization. To annotate features, the accurate mass and tandem mass spectrometry data of each compound were compared with relevant information retrieved from the Human Metabolome Database (HMDB). Precursor and fragment ions were allowed a mass tolerance of 5 and 330 ppm, respectively. Only candidate metabolites with fragmentation scores meeting or exceeding 25 were considered for annotation.

### Statistical analysis

Statistical analyses were performed using GraphPad Prism (La Jolla, CA, USA). Data are presented in terms of the mean ± standard deviation values. Between-group differences were analyzed using the one-way analysis of variance test followed by the Tukey post hoc test and correction. The Pearson correlation analysis was used and performed to analyze the correlations between the study variables (number of rats per group = 4 or 5). A p-value of <0.05 was considered to be statistically significant. Metabolomic data were analyzed using MetaboAnalyst (version 5.0).
